# Increased drug resistance of meticillin-resistant *Staphylococcus aureus* biofilms formed on a mouse dermal chip model

**DOI:** 10.1099/jmm.0.000461

**Published:** 2017-04-28

**Authors:** Shiro Jimi, Motoyasu Miyazaki, Tohru Takata, Hiroyuki Ohjimi, Sadanori Akita, Shuuji Hara

**Affiliations:** ^1^​ Central Laboratory for Pathology and Morphology, Faculty of Medicine, Fukuoka University, Fukuoka, Japan; ^2^​ Department of Pharmacy, Fukuoka University Chikushi Hospital, Fukuoka, Japan; ^3^​ Department of Oncology, Hematology and Infectious Diseases, Faculty of Medicine, Fukuoka University, Fukuoka, Japan; ^4^​ Department of Plastic, Reconstructive and Aesthetic Surgery, Faculty of Medicine, Fukuoka University, Fukuoka, Japan; ^5^​ Department of Plastic Surgery, Wound Repair and Regeneration, Faculty of Medicine, Fukuoka University, Fukuoka, Japan; ^6^​ Department of Drug Informatics, Faculty of Pharmaceutical Sciences, Fukuoka University, Fukuoka, Japan

**Keywords:** biofilm, dermal chip, drug resistance, *in vitro*, MRSA

## Abstract

**Purpose:**

Meticillin-resistant *Staphylococcus aureus* (MRSA) biofilm formation in humans is of serious clinical concern. Previous *in vitro* studies have been performed with biofilms grown only on inorganic substrates; therefore, we investigated the vancomycin (VCM) resistance of MRSA biofilms grown on skin tissue.

**Methodology:**

We established a novel tissue substrate model, namely MRSA grown on segments of mouse skin tissue (dermal chips, DCs), and compared its resistance capacity against VCM with that of MRSA biofilms grown on plastic chips (PCs).

**Results/Key findings:**

For one MRSA isolate, we found that the VCM MIC was identical (1.56 µg ml^−1^) for planktonic cultures and for biofilms-formed on PCs (PC-BF), although the minimum bactericidal concentration (MBC) increased to 6.25 µg ml^−1^ in PC-BF. On the contrary, the MIC and MBC for biofilms formed on DCs (DC-BF) significantly increased (25 and 50 µg ml^−1^, respectively). Furthermore, the minimum biofilm-eradicating concentration was higher for DC-BF (100 µg ml^−1^) than for PC-BF (25 µg ml^−1^). Using six MRSA strains, we found that in PC-BF, the c.f.u. number decreased with increasing VCM concentration, whereas in DC-BF, it greatly increased until the MIC was reached, accompanied by the formation of large colonies, thicker bacterial walls and the presence of many mitotic cells.

**Conclusion:**

Our results indicate that the VCM resistance of MRSA was greater in DC-BF. We conclude that DCs may provide a specific environment for MRSA that enhances bacterial growth under cytotoxic VCM concentrations, and might be useful for the study of skin wound infections and the effects of antimicrobial drugs.

## Introduction

*Staphylococcus aureus* is a Gram-positive, human commensal bacterium, commonly found on the skin of healthy people. Over the last half century, these bacteria have developed resistance to antimicrobial agents commonly prescribed in hospitals. Meticillin-resistant *S. aureus* (MRSA) is phenotypically associated with the presence of the penicillin-binding protein 2a (PBP2a) [1]. PBP2a has a significantly lower affinity for β-lactam antibiotics, which permits cell wall synthesis during antibiotic treatment, whereas wild-type penicillin-binding proteins are inactivated when bound to β-lactams. PBP2a is encoded by the *mecA* gene, which is located in *mec*, a foreign DNA region, and is carried on a distinct mobile genetic element (*SCCmec*) [1].

The bactericidal action of vancomycin (VCM) results predominantly from inhibition of cell wall biosynthesis [2]. VCM preferentially prevents the integration of*N*-acetylmuramic acid (MurNAc)- and *N*-acetylglucosamine (GlcNAc)-peptide subunits into the peptidoglycan matrix, which is the main structural component of the cell wall of Gram-positive bacteria, including MRSA. The hydrophilic moiety of VCM is able to form hydrogen bonds with the terminal d-alanyl-d-alanine moieties of the MurNAc-/GlcNAc-peptides, preventing their incorporation into the peptidoglycan matrix. Although new antimicrobial agents have been developed, VCM is still widely used against MRSA infection [3–5].

*In vivo* biofilms exhibit a drug-tolerant nature and show nonspecific resistance against a multiple spectrum of antibiotics. Biofilms are formed on indwelling foreign bodies, such as catheters, and on necrotic tissue in wounds. Extracellular polysaccharides (EPS) form the major component of the biofilm matrix [6], which decreases drug permeability, thereby leading to drug tolerance and the appearance of persisters or small colony variants due to biological stress [7–9]. However, the exact mechanism of the 10–1000-fold increase in drug tolerance observed in biofilms is still unclear.

In immunocompromized patients, especially those suffering from skin barrier distortion, *S. aureus* can invade the skin, attach to the extracellular matrix using adhesive matrix molecules (MSCRAMMs) present on their surface and form a biofilm [6, 10–12]. This biofilm contains extracellular substances such as EPS that act not only as structural components of the biofilm, but also confer drug tolerance on the bacteria and the capacity to escape the host immune responses [6].

Biofilm formation by MRSA in the human body is of serious clinical concern. It is known that severe MRSA infection in the clinic is difficult to eradicate, leading to frequent relapse. Previous *in vitro* studies of biofilm formation were performed with artificial substrates, such as plastic, silicon and glass. However, the biological behaviour of bacteria on these substrates might differ from that in tissue. We therefore established a novel substrate to be used as a model for biofilm formation on biological tissue, and investigated its effect against VCM.

## Methods

### Bacteria

For the present study, we used an established MRSA strain (ATCC 33591). One hundred and seventy-four clinical samples of MRSA were isolated in Fukuoka University Hospital, one of which (OJ-1) was from an ulcerated wound [13] and four (T12, T34, T41 and T144) were from blood [14]. These particular bacterial isolates were selected because of their superior ability to form stable biofilms. MRSA samples were stored in a deep-freeze, and upon thawing were incubated on tryptic soy agar (TSA) (Becton Dickinson) containing 0.5 % NaCl. Upon colony formation, one colony was inoculated in 5 ml tryptic soy broth (TSB) (Becton Dickinson) in a 12 ml plastic test tube with a screw cap (Sarstedt) at 37 °C. Cultures that achieved stable growth were subsequently cultured on agar, and the colonies formed were stored at 4 °C and used for experiments within 1 month.

### Preparation of dermal chips (DCs)

**Fig. 1.**
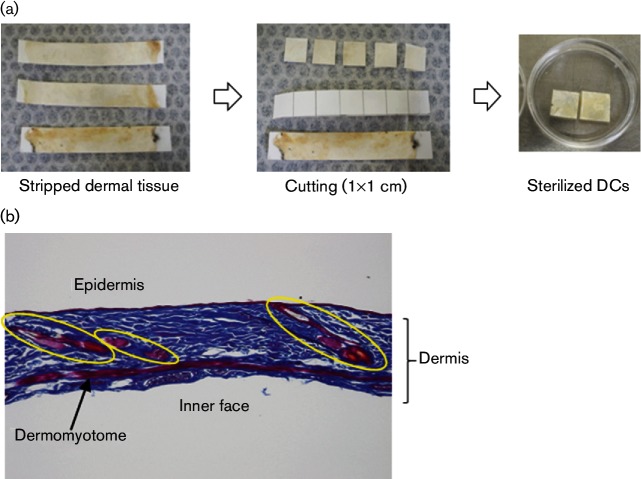
Preparation and structure of DCs. (a) DCs were prepared from extended mouse skin on cardboard, stored at 4 °C and used for experiments within 3 months. (b) The DC morphology was analysed by Masson Trichrome staining. Hair roots and sebaceous glands (indicated by yellow circles), the dermomyotome and other skin appendages were well preserved in the DC.

All animal experiments carried out in this study received prior approval from the animal experiment approval committee of Fukuoka University Animal Center (approval number 1210608). Female C57BL/6 N mice (Japan SLC) were used. Under anaesthesia with Somunopentyl (Kyoritsu-Seiyaku), depilation was performed using a commercial hair remover. Animals were sacrificed by cervical dislocation and their complete skin tissue was obtained. After removal of excess fat and muscle with tweezers, the internal face of the skin was spread and adhered on cardboard, and fixed in 99 % ethanol for 24 h. The fixed skin was then dried on a clean bench with airflow. The skin sheet on the cardboard was cut into 1×1 cm pieces and sterilized by ethylene oxide gas (Fig. 1a). These pieces, referred to as DCs, were stored at 4 °C and used within 3 months. The skin structure was well preserved in the DCs (Fig. 1b).

### Preparation of biofilms on chips

One colony, which was grown on TSA, was inoculated in TSB in a 12 ml tube at 37 °C until its optical density at 578 nm reached the value of 0.57. Subsequently, a bacterial solution diluted 1000-fold in TSB was used for biofilm formation on DCs and plastic chips (PCs) cut from an overhead projector film sheet (3M). Briefly, the DC and PC pieces (1×1 cm) were immersed in 10 ml bacterial solution in a 12 ml tube, and cultured on a rotary shaker (NR-2; Taitec) at 37 °C under shaking (150 r.p.m.) for 24 h to obtain a uniform biofilm on their surfaces. After incubation, the biofilms were washed thrice in 10 ml 0.01 M PBS solution (pH 7.4) to remove the planktonic (PK) cells. The biofilms formed on DCs and PCs are referred to as DC-BF and PC-BF, respectively.

### VCM exposure

VCM (Sigma-Aldrich) in TSB at 400 µg ml^−1^ was serially diluted up to 0.39 µg ml^−1^. The effects of VCM on the biofilms were evaluated by the following parameters: MIC, minimum bactericidal concentration (MBC) and minimum biofilm-eradicating concentration (MBEC). Bacterial growth upon VCM exposure was evaluated in 12 ml tubes by measuring the optical density at 578 nm. The number of live bacteria was determined by a c.f.u. assay, and was almost identical in PC-BF (6.1±1×10^6^) and DC-BF (6.7±1×10^6^). PC-BF, DC-BF and the same number of PK cells were exposed to different concentrations of VCM at 37 °C for 24 h to obtain the MIC values. Subsequently, 20 µl medium from each tube was blotted on a 1×1 cm filter paper on TSA, incubated for 12–16 h at 37 °C, and the MBC was determined by the appearance of growing colonies around the paper. Additionally, biofilm chips were placed on TSA, incubated overnight at 37 °C, and the MBEC was determined by the appearance of growing colonies around the chips.

### Determination of the number of viable bacteria in the biofilms on chips

The biofilms on chips (PC-BF and DC-BF) were washed with PBS, placed in 2 ml PBS in a Petri dish, mechanically scrubbed with a spatula and were washed thrice with 1 ml PBS each time. The total PBS volume (5 ml) was collected in a culture tube. Histological analysis revealed almost no remaining bacteria on the chips. In the case of biofilms formed on the tube surface, the tube was washed thrice with 10 ml PBS each time, and subsequently 5 ml PBS was added to the tube. The tube with the PBS solution with the biofilms formed on the tube surface or on chips was sonicated on ice at an output level of 2 and a 50 % duty cycle of 30 s (Sonifier 250; Branson Ultrasonics). The bacterial solution obtained was serially diluted 10-fold, and 50 µl each dilution was inoculated overnight on TSA. The number of c.f.u. was calculated by counting the number of colonies formed.

### Biofilm staining

DC-BF were incubated with VCM, fixed in 5 % neutral formalin and embedded in paraffin blocks. Similarly, PK cells were also embedded in a paraffin block. The blocks were cut into 4 µm thin sections and used for histological staining, i.e. haematoxylin–eosin staining and Gram staining. For acid mucopolysaccharide staining, alcian blue (ALB) staining (pH 2.5), toluidine blue staining (pH 2.5) and Fe colloid staining were performed. For neutral mucopolysaccharide staining, periodic acid–Schiff (PAS) staining was performed. Immunostaining was performed using a horseradish peroxidase-labelled anti- *S. aureus* antibody (ViroStat), and diaminobenzidine for developing the signal. For double staining, immunostaining was performed after ALB staining.

### Electron microscopy

DC-BF were incubated with VCM, fixed in 2 % glutaraldehyde in 0.1 M PBS (pH 7.4), post-fixed with 0.5 % OsO_4_ in 0.1 M PBS and dehydrated with acetone. The sample was then embedded in Epon resin, and ultra-thin sections (70 nm) were cut. The sections were stained with uranyl acetate and lead nitrate, and coated with carbon. The ultrastructure of the biofilm was then observed by transmission electron microscopy (100CX; JEOL).

### Morphometrical analysis

In electron micrographs (×15 000 magnification), bacteria possessing a large, round body were chosen for analysis. After importing the images into image analysis software (VH Analyzer ver 2.60; KEYENCE), the area surrounded by the cell wall (W-area) and the cell membrane (M-area) was measured. The diameter of the circles surrounding the W-area and the M-area was calculated by the following formulas: W-diameter (W-dia)=2×√ (W-area/3.14), M-diameter (M-dia)=2×√ (M-area/3.14). Cell wall thickness (CWT) was calculated by the following formula: CWT=(W-dia – M-dia)/2. The surface area lined by the cell wall was calculated as 3.14×W-dia^2^. We measured a total of 80 bacteria per group. For cell division, bacteria containing the metaphase plate were considered as dividing cells; more than 200 cells were analysed per group.

### Data and statistical analysis

Results from two different experimental groups were compared using Student’s *t*-test. *P* values <0.05 were considered to denote statistical significance. Data are expressed as mean values ±se.

## Results

### Experimental procedure

**Fig. 2.**
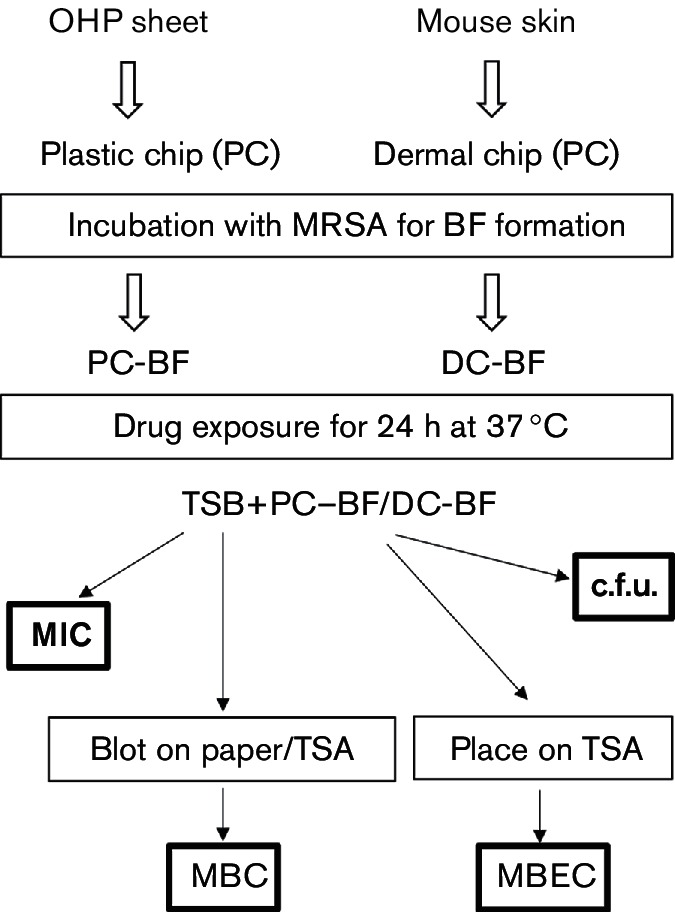
Summary of the experimental procedure.

The basic experimental procedure followed in this study is summarized in Fig. 2.

### Histological structure of DC-BF

**Fig. 3.**
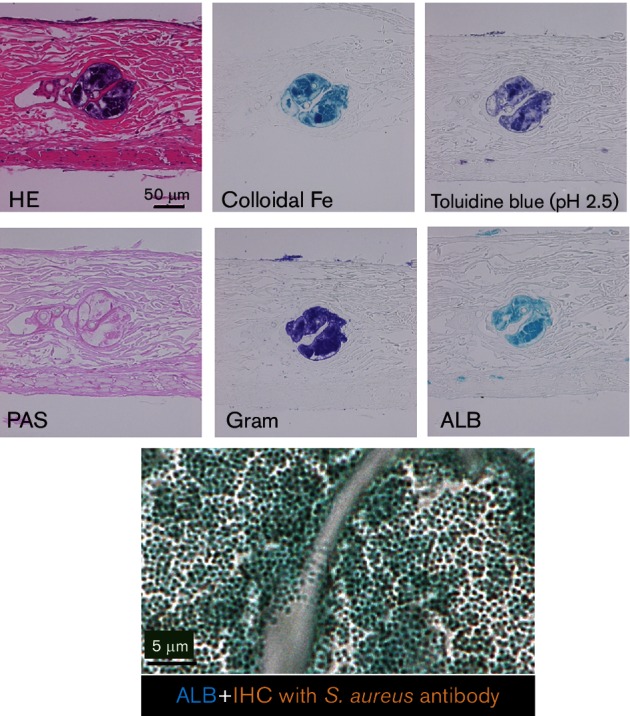
Detection of MRSA colonies and their biofilm matrix on DCs. An MRSA clinical isolate (OJ-1) was used. Colonies were developed inside the internal root sheath of the DCs following 24 h incubation with the bacterial culture. Serial sections were subjected to haematoxylin–eosin (HE) staining, Gram staining and immunostaining using an anti-*S. aureus* antibody. PAS staining for neutral mucopolysaccharide and acidic mucopolysaccharide staining, specifically Fe colloid staining, toluidine blue staining (pH 2.5) and ALB staining (pH 2.5), were also performed. Bacterial cells and EPS were examined by double staining using ALB and *S. aureus* antibody (ALB+IHC). The surrounding matrix (blue) was positive for acidic mucopolysaccharides. Bars, 50 µm in serial staining, 5 µm in double staining.

Serial sections of DC-BF from the OJ-1 sample, containing bacterial aggregates, were examined by different staining methods. Aggregates of different sizes, from small to large, were found in tissue cleavages, such as hair roots and sebaceous glands (Fig. 3). Gram-positive bacterial nuclei were stained with haematoxylin–eosin and were found to be positive for staining with the anti-*S. aureus* antibody as well. The biofilm matrix was detected by mucopolysaccharide staining. PAS staining for neutral mucopolysaccharides failed to show any specific tissue localization. On the contrary, Fe colloid staining, toluidine blue staining (pH 2.5) and ALB staining (pH 2.5) for acidic mucopolysaccharides were positive in bacterial colonies. In addition, double staining with ALB and immunostaining against *S. aureus* were used to examine the relationship between the bacterial cell body and the EPS. The results demonstrate that circular and granular bacteria were assembled together and were surrounded by a biofilm matrix containing acidic mucopolysaccharides.

### Response of PK cells and biofilm chips to VCM exposure

The capacity of OJ-1 bacteria to develop drug resistance was analysed in three different bacterial states: PK cells, PC-BF and DC-BF. The results are shown in Table 1. The MIC for PK cells and PC-BF was 1.56 µg ml^−1^, whereas that for DC-BF was 25 µg ml^−1^. The MBC for PK cells was 1.56 µg ml^−1^, whereas that for PC-BF was 6.25 µg ml^−1^; the MBC for DC-BF was further increased to 50 µg ml^−1^. The MBEC for PC-BF was 25 µg ml^−1^, and that for DC-BF was 100 µg ml^−1^. In summary, the VCM concentration required to kill bacteria was approximately 16-fold and 64-fold higher for PC-BF and DC-BF, respectively, than in PK cells.

**Table 1.** Drug resistance of MRSA in different states The drug-resistance parameters MIC, MBC and MBEC were analysed after exposure to VCM. Similar experiments were performed 12 times, and the modal value is shown.

State	VCM (μg ml^−1^)		
	MIC	MBC	MBEC
PK	1.56	1.56	－
PC-BF	1.56	6.25	25
DC-BF	25	50	100

### Histological appearance of bacterial colonies in DC-BF

**Fig. 4.**
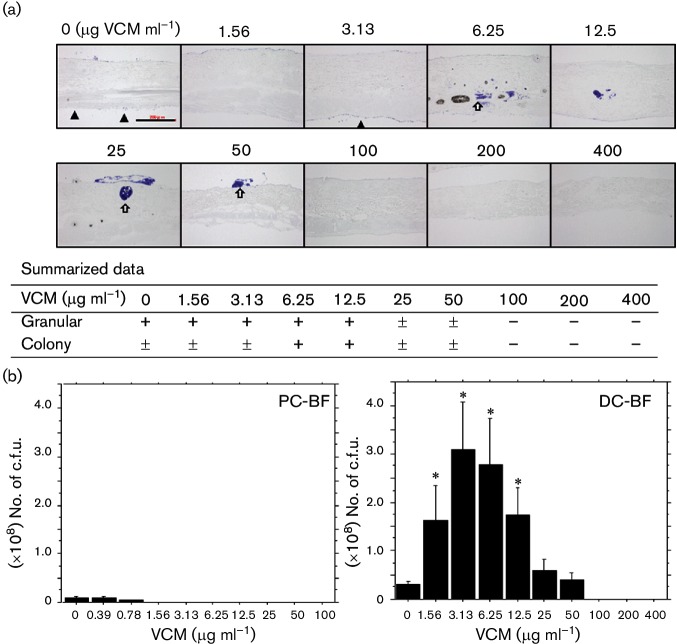
Distribution of OJ-1 in DC-BF exposed to VCM. (a) A granular pattern (indicated by arrowheads) and a colony-forming pattern (indicated by arrows) of bacterial distribution of OJ-1 were observed after 24 h incubation with VCM at different concentrations. The summarized data (*n*=5) are also shown. The extent of bacterial accumulation is indicated as follows: −, negative; ±, occasionally positive; +, frequently positive. Bar, 200 µm. (b) Histograms of c.f.u. values in PC-BF (left panel) and DC-BF (right panel) after exposure to different VCM concentrations. The data shown are mean values ± se. *, *P*<0.05.

After exposure to different concentrations of VCM, DC-BF formed by OJ-1 was subjected to Gram staining. Two distribution patterns were observed (Fig. 4a, upper panel); one showed granular deposition scattered around the tissue surface (indicated by arrowheads) and the other was biofilm-associated colony formation, which was found in tissue cavities (indicated by arrows), such as in hair roots and sebaceous glands. The summarized data (Fig. 4a, lower panel) showed that both the granular pattern and colony formation were observed after VCM exposure up to a concentration of 50 µg ml^−1^. However, large colony formation was more frequent at VCM concentrations of 6.25 and 12.5 µg ml^−1^. No bacteria were found after exposure to more than 100 µg VCM ml^−1^.

### Survival of bacteria in PC-BF and DC-BF after VCM exposure

The c.f.u. assay was performed after exposure of OJ-1 biofilms to VCM. In PC-BF, the c.f.u. value decreased dose dependently with VCM concentration, up to a concentration of 3.13 µg ml^−1^ (Fig. 4b, left panel, Table S1, available in the online Supplementary Material). In contrast, the c.f.u. values in DC-BF increased in a dose-dependent manner with VCM concentrations, up to a concentration of 3.13 µg ml^−1^, at which point they were 10 times greater than those observed without VCM (Fig. 4b, right panel, Table S1). For VCM concentrations higher than 6.25 µg ml^−1^ and up to 100 µg ml^−1^, the c.f.u. values decreased. No living bacteria were detected at VCM concentrations higher than 200 µg ml^−1^. Overall, the number of live cells in DC-BF showed a bell-shaped response against VCM exposure.

**Fig. 5.**
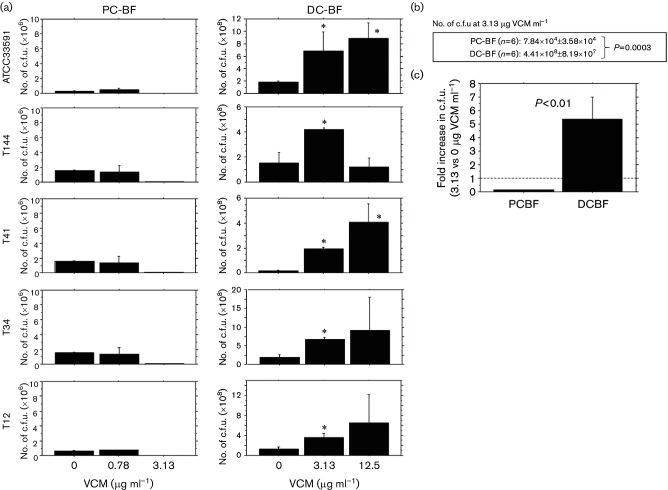
c.f.u. analysis of PC-BF and DC-BF formed by different MRSA 24 h after VCM exposure. (a) PC-BF and DC-BF formed by a MRSA strain (ATCC 33591) and four clinical MRSA isolates were incubated for 24 h with different concentrations of VCM. c.f.u. values for PC-BF (left panels) and DC-BF (right panels) are shown. The data shown are mean values ± se. *, *P*<0.05. (b) Comparison of c.f.u. between PC-BF and DC-BF using six different MRSA. (c) Comparison of the rate of c.f.u. increase upon VCM exposure (3.13 vs 0 µg VCM ml^−1^) between PC-BF and DC-BF using six different MRSA.

To compare the capacity of PC-BF and DC-BF to develop resistance to VCM, we used the ATCC 33591 MRSA strain and four clinical isolates of MRSA (T12, T34, T41 and T144). Based on the previous results obtained using OJ-1, VCM was used at the following concentrations: 0, 0.78 and 3.13 µg ml^−1^ for PC-BF; and 0, 3.13 and 12.5 µg ml^−1^ for DC-BF. In PC-BF, the c.f.u. decreased with VCM concentration in a dose-dependent manner for all MRSA samples (Fig. 5a, left panel, Table S2). On the contrary, in DC-BF, the c.f.u. increased in a dose-dependent manner with VCM concentration for all MRSA samples except for the T144 isolate (Fig. 5a, right panel, Table S2). When the c.f.u. values were compared between PC-BF and DC-BF at 3.13 µg VCM ml^−1^, DC-BF showed greater tolerance in all six MRSA samples including OJ-1 (higher than 10^4^-fold, *P*=0.0003) (Fig. 5b). When the change in c.f.u. between 3.13 and 0 µg VCM ml^−1^ was compared instead, DC-BF responded positively, whereas PC-BF showed a negative response (*P*<0.01) (Fig. 5c and Table S3).

### Morphological alteration of bacteria

**Fig. 6.**
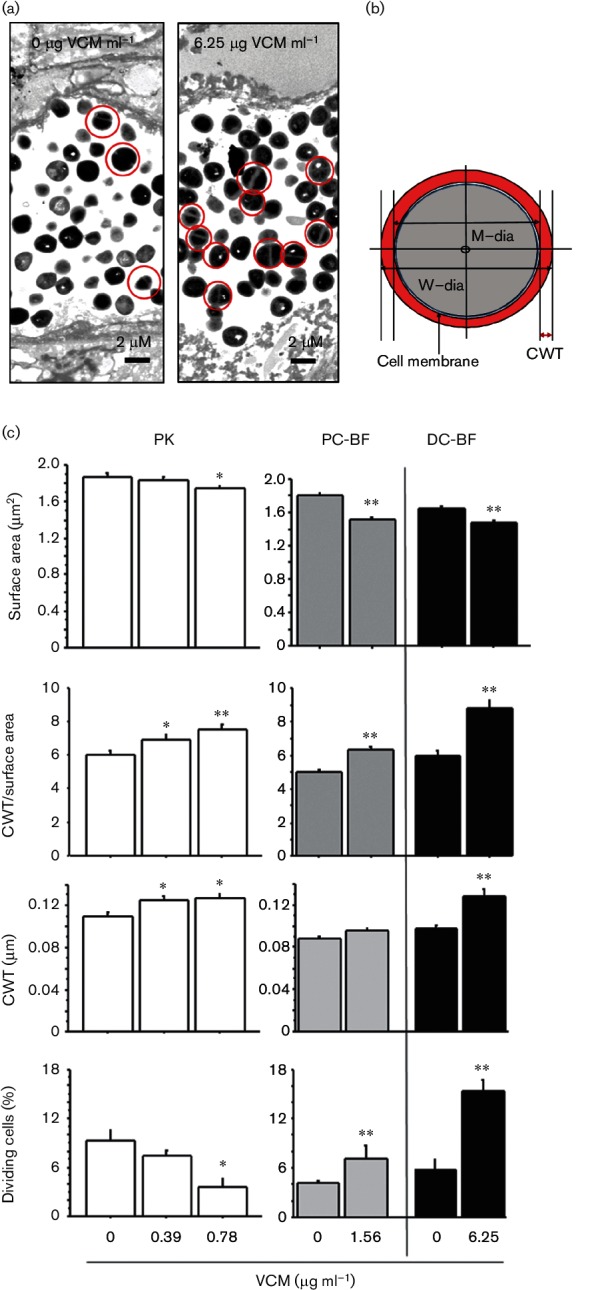
Morphometrical comparison of OJ-1 ultrastructure between different bacterial states. The effects of VCM on different bacterial states, namely PK cells, PC-BF and DC-BF, were assessed by electron microscopy. The cell division rate and several morphometrical parameters of cellular structure were evaluated. (a) Representative electron microscopic images of mitotic bacteria in DC-BF incubated without VCM (left) and with VCM (right). Dividing cells containing the metaphase plate are indicated by red circles. An increase in the number of dividing cells was found in the DC-BF incubated with VCM. Bars, 2 µm. (b) Diagram describing the structural elements used for the morphometrical analysis, including CWT, as described in detail in Methods. (c) Three different states of bacteria (PK cells, PC-BF and DC-BF) after VCM exposure were compared in terms of cell division and structural alterations, including CWT and surface area, calculated by use of the W-dia. The VCM concentrations used were lower than the MIC for each bacterial status. *, *P*<0.05; **, *P*<0.01.

The effects of VCM on different bacterial states of OJ-1 were examined at the ultrastructural level. VCM concentrations less than the MIC for each bacterial state were used as follows: 0.39 and 0.78 µg ml^−1^ for PK cells; 1.56 µg ml^−1^ for PC-BF; and 6.25 µg ml^−1^ for DC-BF. Morphometrical analysis for cell division and cellular structure was performed using electron micrographs (Fig. 6 a, b).

#### Cell division

For PK cells in the log phase, the proportion of dividing cells containing the metaphase plate was 27.3 %, which was significantly higher (approximately threefold) than that of cells in the stationary phase (0 µg VCM ml^−1^). In the absence of VCM, the proportion of dividing cells tended to be lower in DC-BF than in PK cells. In PK cells, the proportion of dividing cells after incubation with VCM was decreased in a dose-dependent manner, and a significant decline was found at 0.78 µg VCM ml^−1^ (Fig. 6c and Table S4). In contrast, the biofilm formed on both chips showed a significant increase in dividing cells after VCM exposure as follows: 1.7-fold in PC-BF and 2.6-fold in DC-BF.

#### Surface area

The surface area of the cell wall changed with bacterial status as follows: PK cells > PC-BF > DC-BF. In all bacterial states, it was significantly decreased after VCM exposure (Fig. 6c, Table S4).

#### CWT

CWT in all bacterial states tended to be greater after VCM exposure; in DC-BF, it was found to be significantly greater after incubation with 6.25 µg VCM ml^−1^ (1.3-fold, *P*<0.0001). These changes became more apparent when CWT was corrected by the surface area (Fig. 6c and Table S4).

## Discussion

Once *S. aureus* enters a host body, its proteinaceous and non-proteinaceous adhesins mediate attachment to the extracellular matrix and cells [6, 10–12]. Then, the bacteria produce extracellular components and form biofilms. In order to detect biofilms within tissues, a method for staining polysaccharides is used, considering that polysaccharides are present in large amounts in biofilms and work as EPS to protect the bacteria from a wide range of stresses, including desiccation, antibiotic penetration and invasion by phagocytic cells [6]. In a previous *in vivo* study, we established a novel method to detect MRSA biofilms formed in the murine liver [14]. The same method was adopted here to detect the biofilm matrix in DC-BF. Biofilm formation accompanied by bacterial aggregation was mainly observed in small tissue cleavages, and the biofilm matrix was rich in acidic mucopolysaccharides. Crystal violet, which is generally used for detecting biofilms on plastic and glass laboratory equipment, could not be used because it stains all tissue elements (unpublished data). As acidic mucopolysaccharides are also present in mast cells, mesothelial cells and goblet cells, caution is needed when identifying biofilms in tissues by this method. However, simultaneous evaluation of Gram staining and morphological characteristics can increase the degree of certainty of biofilm identification. In this study, ALB staining showed that bacterial aggregates in the dermal tissue formed an acidic mucopolysaccharide-associated biofilm.

To date, many studies have shown that biofilms develop higher drug resistance than PK cells [15, 16]. The present study showed that biofilms formed in dermal tissue exhibited higher resistance to VCM than those formed on plastic substrates. Bacterial infections observed in clinical practice are associated with both foreign body infections and tissue infections [17]. However, for *in vitro* experiments, plastic substrates are generally utilized, possibly owing to these being readily available. To the best of our knowledge, this study is the first to use a tissue substrate for biofilm formation *in vitro*.

The capacity of MRSA to develop VCM resistance (MIC, MBC and MBEC) was analysed using the clinical isolate OJ-1, which has a superior ability to form biofilms as shown by our previous studies [13, 14]. In comparison to cells in the PK state, the VCM concentration required to kill bacteria was increased by 16-fold in PC-BF and by 64-fold in DC-BF, indicating that DC-BF exhibits high VCM resistance. All of the properties associated with VCM resistance were increased in the biofilm state, especially in DC-BF. The VCM MIC was 1.56 and 25 µg ml^−1^ for PC-BF and DC-BF, respectively. The maximum number of bacteria in a biofilm formed on a plastic substrate (plastic tube) was 5×10^8^ c.f.u., which was more than 70-fold higher than that detected in DC-BF (Fig. S1), whereas the MIC only increased to 12.5 µg ml^−1^ and was still less than that in DC-BF. These results indicate that both types of biofilm (on plastic and tissue) are resistant to VCM, but their residence capacity is different; the resistance capacity is increased by some mechanism when the biofilm is formed on tissue.

To analyse the differences between PC-BF and DC-BF, we examined the biofilm bacterial states using the OJ-1 isolate. Under different concentrations of VCM, bacterial c.f.u. analysis showed that PC-BF and DC-BF exhibited a different response. This finding was also supported by experiments using other MRSA strains, including ATCC 33591 and four additional clinical isolates. Overall, the results were similar across the different strains, confirming that the VCM eradication capacity of the bacteria was definitely lower in PC-BF than DC-BF, and the survival response against increasing VCM concentrations was negative for PC-BF and positive for DC-BF. These results indicate that different biofilm substrates have different resistance capacities, and that biofilms formed on tissues showed the greatest drug resistance. It has been shown that persisters, namely tolerant *S. aureus*, remain in biofilm after exposure to antibacterial drugs [7, 8]. However, the mechanism for the generation of persisters is still unclear. Furthermore, it is also known that small colony variants appear as bacterial subpopulations with higher drug-resistance capacity after treatment with antibacterial drugs [9]. Further studies regarding the phenotypic alterations in DC-BF with respect to acquired drug resistance are necessary.

It is known that the peptidoglycan-based cell wall of MRSA is thickened after VCM exposure, possibly owing to a reactive alteration leading to drug resistance [18]. Such changes have also been observed in MRSA after exposure to daptomycin [19]. Moreover, Onyango *et al.* [20] have reported that in addition to changes in the cell wall, the cell sizes of staphylococci are altered after exposure to antibacterial agents; the cells become thicker and smaller after VCM exposure. In our study using OJ-1, in the steady state, size increased in the order of DC-BF<PC-BF<PK. Interestingly, the differences in both parameters were amplified by VCM exposure. These morphological alterations in bacteria might be an avoidance reaction against VCM toxicity. Biofilms, especially DC-BF, may provide a special environment for bacteria to combat antibacterial agents.

An interesting finding of this study was that under various concentrations of VCM, the number of live cells in DC-BF showed a bell-shaped response when the biofilms were exposed to VCM concentrations lower than those considered bactericidal. Wang *et al*. [21] have also reported that the biofilms formed by *Staphylococcus epidermis* after exposure to antibacterial drugs increase in amount, accompanied by an increase in the expression of related genes. This finding was supported by our histological distribution of MRSA colonies in DC-BF exposed to VCM. To confirm the temporal growth enhancement during VCM exposure found in DC-BF (Fig. 4b), mitotic cells containing the metaphase plate were detected by electron microscopy, and were considered as a mitotic index. Analysis of DC-BF exposed to different VCM concentrations showed that the emergence of mitotic cells correlated with the c.f.u. response. These results suggest that bacteria in biofilms under cytotoxic stress may respond by promoting cell growth, and this may be a survival strategy in biofilms.

### Conclusion

In this study, we established a novel experimental device to study biofilms. We found that MRSA biofilms formed in mouse dermal tissue *in vitro* acquired stronger VCM resistance than biofilms formed on plastic substrates. Thus, the VCM concentration required to kill bacteria in biofilms forming on tissues needs to be reconsidered. The exact mechanism underlying the higher drug resistance in DC-BF is still unknown; however, the tissue substrate in DC-BF may provide a distinct environment to MRSA. This unique *in vitro* model might be a useful experimental tool to study skin wound infections, the effects of antimicrobial drugs and bacterial phenotypic alterations after exposure to antimicrobial agents.

## Supplementary Data

106 Supplementary File 1
