# Supplementary material for: Increased drug resistance of meticillin-resistant Staphylococcus aureus biofilms formed on a mouse dermal chip model
Source: J Med Microbiol. 2017 Apr 28;66(4):542–50. doi: 10.1099/jmm.0.000461 (PMC5817199; doi:10.1099/jmm.0.000461)

Supplemental Table 1. Colony-forming unit (CFU) data for plastic chip-biofilm (PC-BF) and dermal chip-biofilm (DC-BF) formed by OJ-1, a MRSA isolate, after exposure to different vancomycin (VCM) concentrations.

The data correspond to the results presented in Figure 4(b).

		PC-BF CFU x10^6			DC-BF CFUx10^7	
VCM (ug/mL)	n	mean	SE	n	mean	SE
**0**	5	8.45	2.74	4	3.16	6.1
**0.39**	5	9.76	182	0	ND	ND
**0.78**	5	3.81	1.28	0	ND	ND
**1.56**	5	0.02	0.02	4	16.46	6.94
**3.13**	5	0	0	4	30.86	9.84
**6.25**	5	0	0	4	27.88	9.27
**12.5**	5	0	0	4	17.34	5.59
**25**	5	0	0	4	5.84	2.28
**50**	5	0	0	4	3.88	1.61
**100**	5	0	0	4	0.17	0.08
**200**	0	ND	ND	4	0	0
**400**	0	ND	ND	4	0	0

ND: not determined

Supplemental Table 2. CFU data for PC-BF and DC-BF formed by the established MRSA strain ATCC33591 and five clinical MRSA isolates (T144, T41, T34, T12, and OJ1), 24 h after exposure to different VCM concentrations.

The data correspond to the results presented in Figure 5(a).

		PC-BF CFU × 10^5			DC-BF CFU × 10^8	
MRSA	VCM (μg/mL)	mean	SE	VCM (μg/mL)	mean	SE
ATCC33591	**0**	3.28	0.38	**0**	1.88	0.18
	**0.78**	4.9	2.4	**3.13**	6.9	2.95
	**3.13**	0.04	0.02	**12.5**	8.85	2.5
T144	**0**	10.25	7.25	**0**	1.5	0.85
	**0.78**	4.13	0.48	**3.13**	4.23	0.13
	**3.13**	1.88	1.08	**12.5**	1.18	0.78
T41	**0**	15.53	0.48	**0**	0.15	0.07
	**0.78**	13.35	9.05	**3.13**	1.93	0.13
	**3.13**	0.87	0.23	**12.5**	4.08	1.48
T34	**0**	4.18	1.93	**0**	2	0.65
	**0.78**	4.4	2.1	**3.13**	6.73	0.58
	**3.13**	1.8	0.2	**12.5**	9.18	8.88
T12	**0**	0.645	0.4	**0**	1.3	0.4
	**0.78**	7.85	0.15	**3.13**	3.63	0.73
	**3.13**	0.13	0.03	**12.5**	6.5	5.7
OJ1	**0**	0.84	0.27	**0**	0.32	0.61
	**0.78**	0.38	0.13	**3.13**	1.73	0.56
	**3.13**	0	0	**12.5**	0.39	0.16

Supplemental Table 3. PC-BF and DC-BF formed by six different MRSA strains were exposed to 3.13 μg/mL VCM for 24 h, and the change in CFU (3.13 vs. 0 μg/mL VMC) was compared between the two biofilms.

The data correspond to the results presented in Figure 5(c).

		PC-BF		DC-BF		
	n	mean	SE	mean	SE	
Fold change (3.13 vs. 0 μg/mL VCM)	6	0.14	0.08	5.36	1.64	($p < 0.01$)

Supplemental Table 4. OJ-1 in planktonic (PK), PC-BF, and DC-BF states were exposed to different VCM concentrations and the following morphometric parameters were determined: dividing cells, surface area (SA), cell wall thickness (CWT), and CWT/SA.

The data correspond to the results presented in Figure 6(c).

	VCM (mg/mL)	Dividing cells (%)			Surface area (SA) (μm^2)			CWT (μm)			CWT/SA		
		mean	SE	P	mean	SE	p	mean	SE	p	mean	SE	p
**PK**	**0**	9.28	1.35	*	1.88	0.04	*	0.11	0.004	*	6.02	0.24	*
	**0.36**	7.5	0.53	ns	1.84	0.03	ns	0.125	0.004	<0.05	6.94	0.25	<0.05
	**0.78**	3.68	0.95	<0.01	1.75	0.03	<0.05	0.127	0.004	<0.05	7.48	0.31	<0.01
**PC-BF**	**0**	4.12	0.33	*	1.82	0.04	*	0.088	0.002	*	5	0.17	*
	**1.56**	7.13	1.59	<0.05	1.52	0.03	<0.01	0.096	0.002	ns	6.34	0.17	<0.01
**DC-BF**	**0**	6.81	1.17	*	1.65	0.02	*	0.097	0.003	*	6.01	0.23	*
	**6.25**	15.37	1.35	<0.01	1.48	0.03	<0.01	0.128	0.007	<0.01	8.82	0.45	<0.01

*: used as reference

Supplemental Figure 1. Relationship between VCM minimum inhibitory concentration (MIC) and CFU of biofilms formed on a plastic substrate (tube).

To prepare biofilms formed on the surface of plastic tubes with different amounts of OJ-1 ($6 \times 10^6 \sim 5 \times 10^8$ CFU), different amounts (0.25, 0.5, 1.0, 2.0, 3.0, 4.0, and 5.0 mL) of bacterial solution (1000 times dilution of a solution with OD = 0.57) were dispensed in a 12-mL plastic tube, and incubated at 37 °C for 24 h. Subsequently, the biofilm was washed with PBS and exposed to different amounts of VCM. The VCM MIC was obtained for each biofilm. As can be seen, the change in MIC with the biofilm amount is not very large or steep.

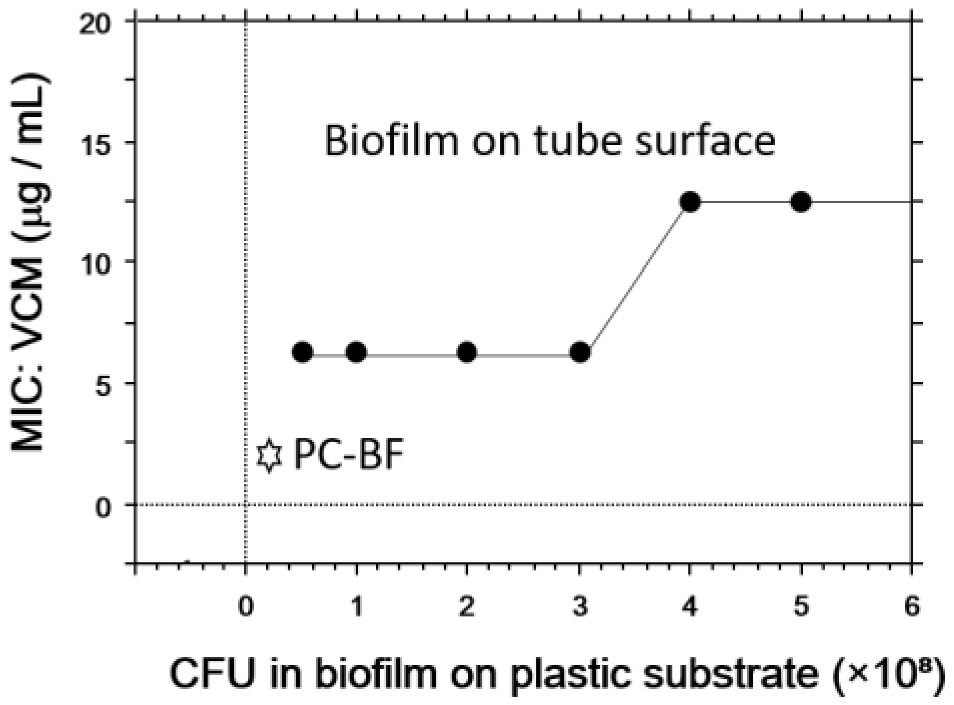

Supplement: Supplementary File 1 [file jmm-66-542-s001.pdf]
